# More 50+ Workers Means More 50+ Policy—Until it Doesn’t. The Non-Linear Relation Between Proportion of Older Workers and Implementation of Policies for Older Workers

**DOI:** 10.1177/07334648231214900

**Published:** 2023-11-29

**Authors:** Jelle Lössbroek, Gerben Hulsegge

**Affiliations:** 1 Netherlands Interdisciplinary Demographic Institute-KNAW, 3647 University of Groningen, The Hague, The Netherlands; 2 Netherlands Organisation for Applied Scientific Research, 2859 TNO, Leiden, The Netherlands

**Keywords:** international, older workers, personnel policies, sociology, training

## Abstract

Personnel policies specifically for older workers can benefit both the older workers and their organization. It is often assumed that a higher percentage of older workers in an organization is associated with *more* policies for older workers. We hypothesize that policies accommodating older workers, such as extra leave or a reduced workload, become unfeasible if the proportion of older workers is high. We pooled data from five datasets to study eleven older-worker policies in 7330 Dutch establishments. The results show that the number of implemented personnel policies for older workers is highest in establishments where 30-50% of the workers are 50 years and older. The number of implemented policies is lower in establishments with more older than younger workers. This pattern is found for most phasing out policies.

**What this paper adds**

• Shows that the prevalence of policies for older workers peaks at 30-50% of older workers and declines with higher percentages of older workers
• Indicates that this trend is visible for highly diverse policies for older workers
• Rejects the notion that in establishments with a majority of older workers, older-worker policies are made available for all workers

**Applications of study findings**

• Researchers studying the influence of personnel composition on policies should not assume that this influence is linear
• Establishments facing an increase in older workers should explore alternatives to existing older-worker policies
• Unions should take into account that further labor force aging may reduce rather than entrench existing policies for older workers

## Introduction

Due to the aging of populations, organizations in many European countries face staff shortages and personnel with changing needs. Supporting personnel to work until a higher age is thus important for many organizations. Hence, they often implement policies specifically for older workers, aimed at either phasing out or at activating personnel. These can have benefits, such as higher job satisfaction (Visser et al., 2021), higher productivity or work ability (Aitken & Singh, 2023; Morelock et al., 2017), delayed retirement (Picchio & Van Ours, 2013), and (via a more positive intergenerational workplace climate) better self-perceived aging, work engagement, and lower turnover intentions (Tybjerg-Jeppesen et al., 2023).

Previous studies indicate numerous reasons why having more 50+ workers could induce more policies for older (aged 50+) workers. The urgency is higher as more work is done by older workers; marginal administrative costs are lower if they can be spread over more workers; there are more opportunities for informal arrangements to be formalized into policy; and personnel representatives are more likely to advocate for the desires of the older workers (Fleischmann et al., 2015; Frerichs et al., 2012; Van Dalen et al., 2015).

We argue that this relationship is different for different kinds of personnel policies. Some personnel policies for older workers try to strengthen their position and increase the job fit. These “developmental” (Van Dalen et al., 2015) or “activating” policies (Lössbroek et al., 2019) comprise training, flexible working hours, and adaptation of tasks. We hypothesize that activating policies are more likely to be implemented if there are relatively many older workers. Other policies offer extra comfort to older workers, for example, by putting an age limit on irregular working hours, offering extra leave, or reducing their workload. In teams with many older workers, these may place an undue demand on younger colleagues, and excluding too many people from certain tasks can be expensive (Streb et al., 2008). For such “phasing out” policies, we expect that implementation peaks at a certain proportion of older workers, and is lower in establishments with a high proportion of older workers. Two studies implicitly acknowledge—but did not directly test—that the share of policies for older workers decreases when the proportion of older workers passes a certain threshold (Lössbroek et al., 2019; Turek et al., 2020). Therefore, this study analyses the central research question: how is the proportion of older workers related to the implementation of policies for older workers? We test two hypotheses.

Hypothesis 1 A higher proportion of older workers increases the implementation of activating policies.

Hypothesis 2 A higher proportion of older workers increases the implementation of phasing out policies, until a threshold, after which it declines.

## Data and Methods

To ensure that our results are not driven by specific properties of a single dataset, we combine five datasets: ASPA (Activating Senior Potential of Ageing), DLDP (Dutch Labour Demand Panel), ESWS (European Sustainable Workforce Survey), NES (NIDI Employer Survey), and NEWS (Netherlands Employers Work Survey). Each data was collected at the level of the establishment (if part of a larger organization), rather than the entire organization. Response rates were in line with or higher than other surveys at the organizational level (Fleischmann et al., 2015). Usually HR managers and directors filled in the survey. DLPD, NES, and NEWS only contain Dutch establishments; ASPA and ESWS also surveyed establishments in other European countries. For consistency, we analyse only Dutch establishments. Table 1 presents more information on data collection.

**Table 1.** Description of Data Collection per Dataset.

	Year	# Establishments	Number of Workers	Response	Mode	Codebook
ASPA	2009	1077^a^	10+	23%	Paper, web, and telephone	(Conen et al., 2011)
DLPD	2017–18	2712	5+	53%	Web and telephone	(SCP, 2018)
ESWS	2015–16	50^a^	20+	Up to 20%	Paper and web	(Van der Lippe et al., 2016)
NES	2017	1358	10+	23%	Paper and web	^
NEWS	2021	4719	1+	33%	Web	(Hulsegge et al., 2021)

*Note.* ASPA = Activating Senior Potential of Ageing in Europe; DLPD = Dutch Labour Demand Panel; ESWS = European Sustainable Workforce Survey; NES = NIDI Employer Survey; NEWS = Netherlands Employers Work Survey; UK = United Kingdom. ^ For NES, no codebook exists.

^a^These are the numbers for Dutch establishments. In total, ASPA contains 6285 establishments; ESWS contains 259 establishments.

### Measurements

This section presents the harmonized measurements; the original measurements can be found in Supplements S1–S7.

We analyse all eleven policies for older workers that are present in multiple datasets. Three concern activating policies: training, flexible working hours and adaptation of tasks. Eight concern phasing out policies: part-time retirement, reduced working hours, early retirement, extra leave, reduced workload, demotion, ergonomic measures, and an age limit to irregular work. Proportion of older workers ranges from 0 to 1; we also divide this into six categories: <10%, 10–19%, 20–29%, 30–39%, 40–49%, and ≥50% older workers. Number of workers is truncated to 5000 as this variable is heavily right-skewed; also, establishments with fewer than 5 workers are omitted as percentages are not meaningful for such small organizations. Establishment structure is dichotomized as 0 “Part of larger organization” and 1 “Independent establishment.” Other covariates are the proportion of female workers, the proportion of temporary workers and dummy variables for sector and dataset (Lössbroek et al., 2019). Descriptive statistics are presented in Table 2 and Table 3.

**Table 2.** Descriptive Statistics.

		All Establishments		Establishments With 50%+ Older Workers	
		*N* = 7330		*N* = 817	
		Mean	SD	Mean	SD
Policy	Proportion of 50+ policies implemented	.29	.22	.32	.23
Proportion	Older workers	.22	.19	.61	.12
	Older workers = 0–10%	.22		.00	
	Older workers = 10–20%	.22		.00	
	Older workers = 20–30%	.21		.00	
	Older workers = 30–40%	.14		.00	
	Older workers = 40–50%	.10		.00	
	Older workers = 50%+	.11		1.00	
	Female workers	.40	.30	.42	.30
	Temporary workers	.24	.24	.16	.21
Type	Independent establishment	.60		.61	
	Number of workers	183.26	514.07	188.06	512.72
Sector	Services	.39		.28	
	Education & science	.11		.15	
	Government	.04		.10	
	Health care	.10		.12	
	Manufacturing	.29		.28	
	Transport	.06		.08	
Source	ASPA 2009	.13		.09	
	DLDP 2018	.16		.32	
	ESWS 2016	.01		.02	
	NES 2017	.17		.29	
	NEWS 2021	.53		.28	

*Note.* All variables range from 0–1 except number of workers (5–5000).

**Table 3.** Mean Values by Data Set.

		ASPA	DLPD	ESWS	NES	NEWS
Policy	Part-time retirement	.51	.23	.59	.29	.19
	Reduced working hours	.38	.41	.67	.35	.45
	Training	.15	.30	.10	.54	.26
	Early retirement	.56	.21	.51	.30	
	Extra leave	.52	.67	.81	.56	.49
	Reduced workload	.38	.36	.41	.27	.31
	Demotion	.08	.10	.35	.13	.08
	Ergonomic measures	.45		.16	.63	.25
	Age limit irregular hours	.27	.25		.30	.22
	Flexible working hours	.45			.62	
	Adaptation of tasks		.28			.18
	All policies combined	.37	.27	.46	.38	.24
Proportion	Older workers	.23	.34	.32	.32	.19
	Older workers = 0–10%	.17	.12	.08	.08	.31
	Older workers = 10–20%	.23	.13	.24	.15	.26
	Older workers = 20–30%	.26	.18		.19	.22
	Older workers = 30–40%	.16	.18	.39	.21	.10
	Older workers = 40–50%	.11	.17		.17	.05
	Older workers = 50%+	.07	.22	.29	.20	.06
	Female workers	.35	.40	.39	.43	.40
	Temporary workers	.13	.17	.18	.14	.33
Type	Independent establishment	.66	.65	.29	.74	.52
	Number of workers	380.41	77.11	679.41	334.04	112.62
Sector	Services	.26	.41	.20	.31	.44
	Education & science	.16	.09	.10	.15	.10
	Government	.13	.07		.04	.02
	Health care	.06	.08	.16	.19	.09
	Manufacturing	.35	.25	.39	.29	.28
	Transport	.04	.09	.14	.02	.07
	*N*	983	1196	49	1216	3886

*Note.* ASPA = Activating Senior Potential of Ageing in Europe; DLPD = Dutch Labour Demand Panel; ESWS = European Sustainable Workforce Survey; NES = NIDI Employer Survey; NEWS = Netherlands Employers Work Survey.

### Estimation Strategy

To estimate the average policy implementation, multivariate linear regression adjusted for the abovementioned covariates is used. We analyse all policies separately, all combined, and the activating and phasing out policies combined. In additional regression models, we add the quadratic term of the proportion of older workers to test the potential non-linear relationship.

We perform numerous additional analyses to test the robustness of our results. First, to explore where a possible “peak” lies, we use a spline regression estimating the proportion of implemented policies (combining all policies). Second, establishments might have “general” policies in place for all workers, for example, by offering everyone flexible working hours or training. We expect that such general policies are offered more often in the establishments with many older workers (i.e., >50% is older), replacing the policies for older workers. Third, it is possible that the absolute rather than the relative number of older workers is most important. Therefore, we replicate the analyses using the absolute number of older workers as predictor. Fourth, given that personnel policies may be more often implemented in larger organizations, we replicate the analyses after splitting the data based on establishment type (independent vs. part of larger organization) and number of workers.

## Results

**Figure 1.**
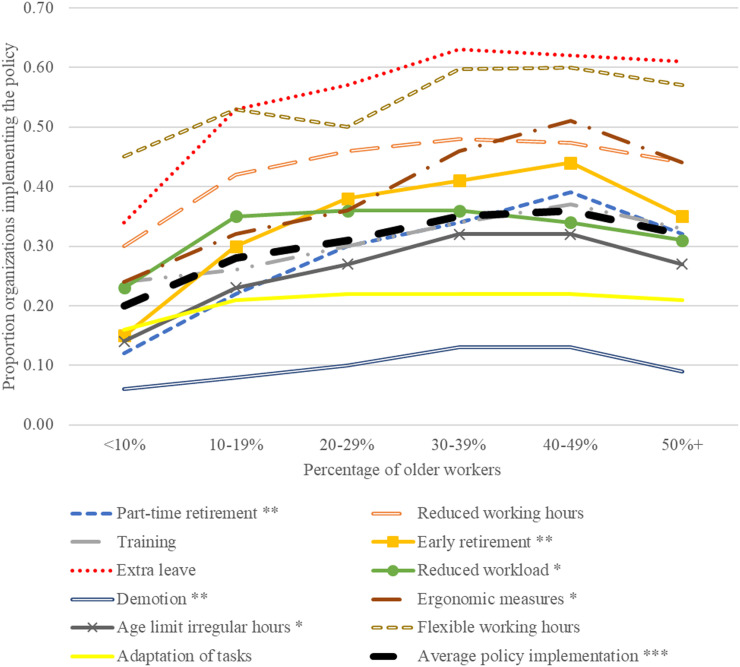
Proportion of organizations implementing a policy for older workers, by the percentage of older workers. Two-sided t-tests between the 50%+ category and the top of the curve show statistically significant differences at *p < .05, **p < .01. ***p < .001.

Figure 1 shows that an increase of the number of policies for older workers coincides with a higher proportion of older workers—until about half or more are older workers. For all eleven policies, policy implementation is lower in the final category (50% or more older workers) than at the peak. This difference is statistically significant (at *p* < .05) for 6 out of 8 phasing out policies. Establishments with less than 10% older workers implement 20% of the policies, which gradually increases to 36% among establishments with 30–50% older workers, and then declines to 32% in establishments with a majority of older workers. The peak is about 4 percentage points higher than the 50%+ category, which is a relevant difference given that the average policy implementation is 30%. Table 4 shows the regression coefficients for the proportion of older workers, adjusted for the control variables. The same pattern emerges. As hypothesized, the policy implementation for phasing out policies is lower in the highest category of older workers compared to the peak for 4 phasing out policies as well as for the composite measure. This confirms Hypothesis 2. The pattern is not visible for the individual activating policies, but it is present for the composite measure. As the results are mixed, we reject Hypothesis 1. Separate models using the linear and quadratic term of proportion of older workers (presented in the right segment of Table 4) also show that for each policy, the quadratic term is negative and larger than the linear term, indicating this pattern for all eleven policies.

**Table 4.** Regression Coefficients for Policies for Older Workers by the Proportion of Older Workers.

	Age Distribution as Categorical Variables, Using 50%+ as the Reference Category					Age Distribution as Continuous Variables		
	0–10%	10–20%	20–30%	30–40%	40–50%	Prop 50+	Prop 50+^2^	*N*
Combined								
All policies	−.07***	−.01	.00	.03**	.03**	.49***	−.56***	7330
Activating policies	.01	.03	.02	.03*	.03	.17***	−.27***	7191
Phasing out policies	−.11***	−.03**	−.01	.02*	.03*	.71***	−.77***	7329
Activating								
Training	.01	01	.02	.03	.03	.16*	−.23*	7177
Flexible working hours	−.01	.04	.00	.05	.03	.35	−.47	2182
Adaptation of tasks	.02	.04	.03	.02	.01	.08	−.18	4949
Phasing out								
Part-time retirement	−.13***	−.06***	−.02	.01	.04**	.76***	−.78***	7253
Reduced working hours	−.16***	−.05*	−.01	.02	.02	1.03**	−1.16*	7174
Early retirement	−.22***	−.11***	−.04	.01	.05*	1.28***	−.1.36***	3361
Extra leave	−.18***	−.04*	−.02	.01	.00	.97***	−.99***	7184
Reduced workload	−.06**	.04*	.04*	.04*	.03	.61***	−.79***	7178
Demotion	−.00	.01	.02	.04*	.03*	.18***	−.24***	7173
Ergonomic measures	−.04	−.01	−.02	.02	.02	.33***	−.39***	6111
Age limit irregular hours	−.10***	−.03	−.01	.04	.03	.71***	−.78***	7099

*p < .05, **p < .01. ***p < .001.

Each row represents two models, one containing the 10% groups, using 50%+ as the reference category, and one model containing proportion of 50+ workers linearly and quadratically. All models include control variables for proportion of female workers, proportion of temporary workers, number of workers, sector, and data source. Differences in *N* are mainly due to some items not being asked in all five data sources. Full Tables with regression coefficients and standard errors of all variables can be found in Supplement S13a–3e.

## Sensitivity Analyses

The spline regression analysis indicates the peak of personnel policies for older workers is between a third and a half; for establishments with more older workers, implementation of such age-specific policies is lower (see Supplement S8).

We can test the “generalisation” argument for five policies: reduced working time, training, extra leave, ergonomic measures, and flexible working hours. For each policy, organizations with most older workers are not more likely to implement such policies for all workers, implying that policies for older workers are not replaced by general policies (see Supplement S9).

The analysis using absolute number of older workers shows a consistent increase of implemented policies as the absolute number of older workers increases, probably driven by the fact that especially large establishments have more older workers (see Supplement S10).

The fourth sensitivity analysis shows that the influence of age composition was weaker in independent establishments than in establishments that are part of a larger organization, and is weaker when smaller establishments are excluded (see Supplement S11-S12).

## Discussion and Conclusion

In response to an ageing workforce, organizations implement various types of personnel policies specifically for older (50+) workers. We show that there is an “optimal proportion” of older workers for policy implementation, and that this optimal proportion is not in the establishments with the highest percentage of older workers.

We find, based on multivariate linear regression and spline regression, that the implementation of phasing out policies for older workers peaks at a certain proportion of older workers and then falls. Organisations with 30%–50% older workers have the most older worker policies. This is in line with our hypothesis for “phasing out” policies. For the activating policies, no such peak was found, confirming our expectations. While we were able to study 11 different policies, it is possible that other policies are used by establishments with many older workers. They may focus on policies more directed at increasing participation of older workers, including creating a supportive organizational climate, mentoring opportunities, and knowledge sharing practices between younger and older workers (Chen & Gardiner, 2019; Lagacé et al., 2023).

For establishments in which more than 50% is an older worker, it may become more appealing to implement flexible working hours, training and adaptation of tasks for all personnel, rather than only for older workers. This could save administrative costs and prevent younger staff from feeling “left out.” We tested this “generalization” argument in the sensitivity analyses for several policies. However, this argument was not empirically supported. Implementation of some policies, including training, was even lower among establishments in which more than 50% was an older worker.

Scientifically, the observed non-linear relationship is in line with the tipping point argument, which is also used to explain how female managers can only improve gender equality when the proportion of female managers passes a certain threshold (Kräft, 2022). Hence, scholars studying the demographic composition of an establishment should not assume linearity and study policies for older workers in their context. Even if older worker policies are beneficial for older workers, they could still have a net negative influence on the organization if they hinder younger colleagues, or overburden financial resources. These negative consequences could lead to policies not being implemented (anymore). Additionally, it is evident that there are sectoral differences in the implementation of policies for older workers. This may be in part due to different age composition, but also because the need for some policies depends on the type of work. Finally, future studies should take a longitudinal approach to disentangle why establishments with many older workers do not implement new policies for older workers, or remove existing policies once their staff ages. Anecdotic evidence suggests both are happening, but more rigorous data is needed.

Societally, our study corroborates earlier work indicating labor market aging has coincided with an increase in policies for older workers (Fleischmann et al., 2015; Van Dalen et al., 2015). However, governments and unions should not assume that further population aging entrenches these policies—on the contrary. Our study implicates it is important to develop new cost-effective strategies for organizations with a large share of older workers to support their older employees without placing an undue demand on younger colleagues.

## Supplemental Material

Supplemental Material - More 50+ Workers Means More 50+ Policy—Until it Doesn’t. The Non-Linear Relation Between Proportion of Older Workers and Implementation of Policies for Older Workers Supplemental Material for More 50+ Workers Means More 50+ Policy—Until it Doesn’t. The Non-Linear Relation Between Proportion of Older Workers and Implementation of Policies for Older Workers by Jelle Lössbroek and Gerben Hulsegge in Journal of Applied Gerontology.

## Data Availability

ASPA, NES, and NEWS data are not publicly available. ESWS data is restricted; access can be requested via https://public.yoda.uu.nl/i-lab/UU01/87ECE1.html. DLPD data is open-access available via https://easy.dans.knaw.nl/ui/datasets/id/easy-dataset:138609. Code is available from the authors upon request (ASPA Employers Survey, 2009, DLPD: SCP, 2018, NES: NIDI-KNAW, 2017; Hulsegge et al., 2021; Van der Lippe et al., 2016).
