# Supplementary material for: More 50+ Workers Means More 50+ Policy—Until it Doesn’t. The Non-Linear Relation Between Proportion of Older Workers and Implementation of Policies for Older Workers
Source: J Appl Gerontol. 2023 Nov 29;43(5):490–6. doi: 10.1177/07334648231214900 (PMC10981204; doi:10.1177/07334648231214900)
Supplement: Supplemental Material - More 50+ Workers Means More 50+ Policy—Until it Doesn’t. The Non-Linear Relation Between Proportion of Older Workers and Implementation of Policies for Older Workers [file sj-pdf-1-jag-10.1177_07334648231214900.pdf]

SUPPLEMENT TO

More 50+ workers means more 50+ policy – until it doesn't.

The non-linear relation between proportion of older workers and implementation of policies for older workers

Lössbroek, Jelle

Hulsegge, Gerben

Harmonisation

S1 Measurement across the five datasets of policies for 50+ workers
S2 Measurement across the five datasets of proportion of 50+ workers
S3 Measurement across the five datasets of number of workers
S4 Measurement across the five datasets of independent establishment
S5 Measurement across the five datasets of sectors
S6 Measurement across the five datasets of proportion of female workers
S7 Measurement across the five datasets of proportion of temporary workers

Additional analyses

S8 Spline regression
S9 General policies across establishments with different age compositions
S10 Predicting 50+ policies using the absolute number of 50+ workers
S11 Predicting 50+ policies in (non-) independent establishments
S12 Predicting 50+ policies excluding the smallest establishments
S13 Full tables for coefficients in Table 2

Supplement S1. Measurement across the five datasets of policies for older workers

Policy	Dataset	Policy per dataset
Part-time retirement	ASPA	Part-time retirement
	DLPD	Possibility to stop working before age 65
	ESWS	Semi-retirement
	NES	Part-time retirement
	NEWS	Part-time early retirement
Reduced working hours	ASPA	Reduction of working time before retirement
	DLPD	Shorter work week
	ESWS	Reduced working hours for older employees
	NES	Reduced work time previous to retirement
	NEWS	Shorter work week / changed working hours on individual basis
Training	ASPA	Training plans for older workers
	DLPD	Stimulating training or course participation by older workers
	ESWS	Training specifically aimed at older employees
	NES	Education for older employees
	NEWS	Stimulate training and/or course participation
Early retirement	ASPA	Early retirement schemes
	DLPD	Possibility to fully stop work before age 65
	ESWS	Early retirement
	NES	Early retirement
	NEWS	-
Extra leave	ASPA	Possibilities of extra leave for older workers
	DLPD	Extra days off for older workers
	ESWS	Additional leave from work or extra days off for older employees
	NES	Extra days off for older workers
	NEWS	Extra days off for older workers
Reduced workload	ASPA	Decreasing the workload for older workers
	DLPD	Task reduction for older workers
	ESWS	Lighter workload for older employees
	NES	Task reduction for older workers
	NEWS	Task reduction
Demotion	ASPA	Reduction in task and salary (demotion)

	DLPD	Stepping down in function and salary for older workers
	ESWS	Demotion
	NES	Stepping down in function and salary
	NEWS	Stepping down in salary/in function (‘demotion’)
Ergonomic measures	ASPA	Ergonomic measures
	DLPD	-
	ESWS	Health-promoting ergonomic facilities specifically aimed at older employees
	NES	Ergonomic measures
	NEWS	Adapting the workplace
Age limit irregular hours	ASPA	An age limit for irregular work / shift work
	DLPD	Exempt older workers from irregular shifts or shift work
	ESWS	-
	NES	Exempt older people from irregular work / shift work / over time
	NEWS	Exempt older workers from irregular working hours and shift work
Flexible working hours	ASPA	Flexible working hours
	DLPD	-
	ESWS	-
	NES	-
	NEWS	Flexible working time
Adaptation of tasks	ASPA	-
	DLPD	A different set of tasks for older workers
	ESWS	-
	NES	-
	NEWS	Adapting the tasks (job crafting)

Note. ASPA = Activating Senior Potential of Ageing in Europe; DLPD = Dutch Labour Demand Panel; ESWS = European Sustainable Workforce Survey; NES = NIDI Employer Survey; NEWS = Netherlands Employers Work Survey

Supplement S2. Measurement across the five datasets of proportion of older workers

	Question phrasing	Answering options
ASPA	What percentage of the employees are 50 years of age or older?<br>………… % (a rough estimate is okay)	Open question, no answering categories.
DLPD	Can you give the division of the staff per January 1 2017 based on age category?<br>Share aged 50-54:<br>Share aged 55 or older:	Open question, no answering categories. We created a sum score for these two questions
ESWS	What percentage of employees in your establishment ... are 50 or older?	*1 None – coded to 0<br>*2 1% to less than 10% – coded to .05<br>*3 10% to less than 20% – coded to .15<br>*4 20% to less than 40% – coded to .30<br>*5 40% to less than 60% – coded to .50<br>*6 60% to less than 80% – coded to .70<br>*7 80% to less than 90% – coded to .85<br>*8 90% to less than 100% – coded to 1<br>*9 All
NES	What percentage of the staff in your organization… is older than 50 years? (a rough estimation is fine)	Open question, no answering categories.
NEWS	What part of the workforce in your establishment is older than 55 years? (you can provide an estimate)	Open question, no answering categories.

Note. ASPA = Activating Senior Potential of Ageing in Europe; DLPD = Dutch Labour Demand Panel; ESWS = European Sustainable Workforce Survey; NES = NIDI Employer Survey; NEWS = Netherlands Employers Work Survey. For NEWS data, ‘older’ refers to 55+; for all other datasets, it refers to 50+.

Supplement S3. Measurement across the five datasets of number of workers

	Question phrasing	Answering options
ASPA	Approximately how many people are currently employed at this establishment? ... Total	Open question, no answering categories.<br>17 establishments did not report total workers, but did report male & female workers. For these establishments, the sum of these two questions was used.
DLPD	How many people are currently employed by this establishment?	Open question, no answering categories.<br>However, the data collection team recoded this into categories to ensure anonymity. For each category, we coded this as the mean value. Hence:<br>5 – 9 employees – coded as 7<br>10-19 employees – coded as 15<br>20-49 employees – coded as 35<br>50-99 employees – coded as 75<br>100-199 employees – coded as 150<br>200 and more employees – coded as 300
ESWS	How many employees are there in your establishment	Open question, no answering categories.<br>6 establishments did not report total workers, but based on the sampling frame used for data collection it was possible to have an indication about the number of workers<br>100-249 employees – coded as 175<br>250 and more employees – coded as 300
NES	How many employees are currently working at (this establishment of) your organization?	Open question, no answering categories.
NEWS	What is the total number of employees in your establishment at January 1 2021? [= year of data collection]	Open question, no answering categories

Note. ASPA = Activating Senior Potential of Ageing in Europe; DLPD = Dutch Labour Demand Panel; ESWS = European Sustainable Workforce Survey; NES = NIDI Employer Survey; NEWS = Netherlands Employers Work Survey.

Supplement S4. Measurement across the five datasets of independent establishment versus part of larger organisation

	Question phrasing	Answering options	
		0. Part of a larger organization	1. Independent establishment
ASPA	Is your establishment… A single independent organisation?	No	Yes
DLPD	Organisation is only located on this address, or also in other establishments	Multiple establishments	Single address
ESWS	How would you describe your establishment?	The headquarters of a company or organization<br>A branch office or unit of a company or organization	An independent company or organisation
NES	Is your organization independent or part (headquarters or establishment) of a larger organization?	Part of a larger organization	Independent organisation
NEWS	How would you describe the structure of your organization?	Headquarters of a Dutch organization with multiple establishments<br>Headquarters of a foreign organization with multiple establishments	Independent (without other establishments)

Note. ASPA = Activating Senior Potential of Ageing in Europe; DLPD = Dutch Labour Demand Panel; ESWS = European Sustainable Workforce Survey; NES = NIDI Employer Survey; NEWS = Netherlands Employers Work Survey.

Supplement S5. Measurement across the five datasets of sectors

	1. Services	2. Education & science	3. Government	4. Health care	5. Manufacturing	6. Transport
ASPA	Wholesale & retail trade<br>Accommodation & food service<br>Information & communication<br>Finance & insurance<br>Real estate<br>Administrative & support services<br>Arts entertainment & recreation<br>Other	Professional, scientific & technical activities<br>Education	Public administration & defense	Health care & social work	Mining, quarrying<br>Manufacturing<br>Electricity, gas supply<br>Water supply, waste management<br>Construction	Transportation & storage
DLPD	Trade, hospitality & maintenance<br>Business services<br>Other services	Education	Government	Care & wellbeing	Industry & agriculture<br>Construction	Transport
ESWS	Financial services<br>Telecommunication	Higher education		Health care	Manufacturing	Transport
NES	Multiple answers<br>Wholesale & retail<br>Hospitality<br>Information & communication<br>Financial services<br>Real estate<br>Business services<br>Other business services<br>Culture, sports & entertainment<br>Other	Education	Public administration & government	Health care & wellbeing	Mineral extraction<br>Industry<br>Energy<br>Water<br>Construction	Transportation & storage
NEWS	Wholesale & retail<br>Hospitality<br>Information & communication<br>Financial institutions<br>Business services / real estate<br>Culture, sports & entertainment / other	Education	Public administration	Health care & wellbeing	Agriculture, forestry & fishing<br>Industry (including minerals & utilities)<br>Construction	Transportation & storage

Note. ASPA = Activating Senior Potential of Ageing in Europe; DLPD = Dutch Labour Demand Panel; ESWS = European Sustainable Workforce Survey; NES = NIDI Employer Survey; NEWS = Netherlands Employers Work Survey

Supplement S6. Measurement across the five datasets of proportion female workers

	Question phrasing	Answering options
ASPA	Approximately how many people are currently employed at this establishment? ... Women<br>Approximately how many people are currently employed at this establishment? ... Total	Open questions, no answering categories.<br>We divided the number of female workers by the number of total workers to arrive at the proportion
DLPD	Number of female employees	Open question, no answering categories.<br>Recoded by data collection team into a percentage.
ESWS	What percentage of employees in your establishment ... are female?	*1 None – coded to 0<br>*2 1% to less than 10% – coded to .05<br>*3 10% to less than 20% – coded to .15<br>*4 20% to less than 40% – coded to .30<br>*5 40% to less than 60% – coded to .50<br>*6 60% to less than 80% – coded to .70<br>*7 80% to less than 90% – coded to..85<br>*8 90% to less than 100% – coded to 1<br>*9 All
NES	What percentage of the staff in your organization is female? (a rough estimate is fine)	Open question, no answering categories.
NEWS	What part of the staff in your establishment is female? (an estimate is fine)	Open question, no answering categories.

Note. ASPA = Activating Senior Potential of Ageing in Europe; DLPD = Dutch Labour Demand Panel; ESWS = European Sustainable Workforce Survey; NES = NIDI Employer Survey; NEWS = Netherlands Employers Work Survey

Supplement S7. Measurement across the five datasets of proportion of temporary workers

	Question phrasing	Answering options
ASPA	Approximately what percentage of your employees are… Holding a fixed-term temporary contract?	Open question, no answering categories.
DLPD	% of employees with a permanent contract	Open question, no answering categories.<br>We computed the proportion of temporary workers by subtracting this number from 1
ESWS	What percentage of employees in your establishment ... have a fixed-term contract?	*1 None – coded to 0<br>*2 1% to less than 10% – coded to .05<br>*3 10% to less than 20% – coded to .15<br>*4 20% to less than 40% – coded to .30<br>*5 40% to less than 60% – coded to .50<br>*6 60% to less than 80% – coded to .70<br>*7 80% to less than 90% – coded to..85<br>*8 90% to less than 100% – coded to 1<br>*9 All
NES	What percentage of the staff in your organization can be seen as 'flexible periphery' (such as temporary contracts, zero-hour contracts, self-employed)? (a rough estimate is fine)	Open question, no answering categories.
NEWS	Can you divide the total staff in your establishment in…Staff with a permanent contract	Open question, no answering categories.<br>We computed the proportion of temporary workers by subtracting this number from 1

Note. ASPA = Activating Senior Potential of Ageing in Europe; DLPD = Dutch Labour Demand Panel; ESWS = European Sustainable Workforce Survey; NES = NIDI Employer Survey; NEWS = Netherlands Employers Work Survey

Supplement S8. The proportion of implemented policies for older workers estimated using spline regression

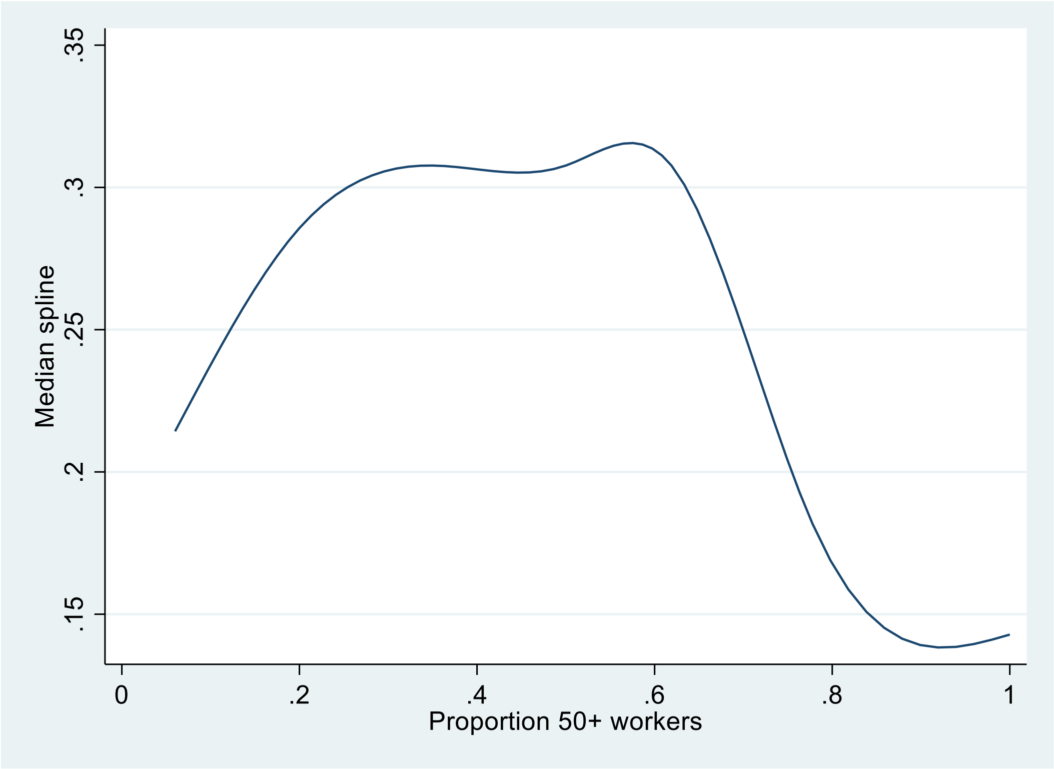

Figure S8. Spline regression predicting proportion of policies for older workers by proportion of older workers

Supplement S8 predicts the proportion of implemented policies for older workers (combining all policies) using a spline regression with seven knots. The peak of personnel policies for older workers is on average around one third, after which it reaches a plateau. For establishments with many older workers, implementation of such age-specific policies is lower. Specifying a higher number of knots yields comparable results; specifying a lower number of knots yields a generally comparable result although then the plateau is far smaller.

Supplement S9. Regression coefficients for general (i.e. non-age-specific) policies across establishments with different age compositions by the proportion of older workers.

	Age distribution as categorical variables, using 50%+ as the reference category					Age distribution as continuous variables		
	0-10%	10-20%	20-30%	30-40%	40-50%	Prop 50+	Prop 50+^{2}	N
Reduced working hours	-.02	.03	.03	.04	.03	.38***	-.50***	4,185
Training	.02	.04*	.04	.01	-.01	.03	-.09	2,960
Extra leave	-.01	-.03	-.02	-.00	.01	-.04	.10	2,866
Ergonomic measures	.03	.09**	.10**	.07	.07	.43**	-.63***	2,886
Flexible hours	-.04	-.02	-.01	-.01	.07*	.15	.-.08	4,190

* *p*<.05, ** *p*<.01. *** *p*<.001
Each row represents two models, one containing the 10% groups, using 50%+ as the reference category, and one model containing proportion of older workers linearly and quadratically. All models include control variables for proportion of female workers, proportion of temporary workers, number of workers, sector, and data source.

Supplement S10. Regression coefficients of policies for older workers, by the absolute number of older workers

	Number of older workers as categorical variable, using 0-4 as the reference category				
	5-15	15-40	40-100	100+	N
**Combined**					
All policies	.07***	.12***	.18***	.25***	7,330
Activating policies	.04***	.08***	.11***	.17***	7,191
Phasing out policies	.09***	.15***	.21***	.29***	7,329
**Activating**					
Training	.04***	.07***	.10***	.17***	7,177
Flexible hours	.05	.12***	.19***	.21***	2,182
Adaptation of tasks	.06***	.07***	.09***	.14***	4,949
**Phasing out**					
Part-time retirement	.09***	.17***	.26***	.37***	7,253
Reduced work time	.15***	.19***	.26***	.35***	7,174
Early retirement	.12***	.19***	.29***	.41***	3,361
Extra leave	.13***	.16***	.21***	.25***	7,778
Decreased workload	.09***	.13***	.14***	.16***	7,772
Demotion	.03*	.07***	.14***	.19***	7,767
Ergonomic measures	.05**	.09***	.16***	.26***	6,111
Age limit irregular	.09***	.19***	.27***	.35***	7,099

* $p<.05$, ** $p<.01$. *** $p<.001$

Each row represents a separate model. All models include control variables for proportion of female workers, proportion of temporary workers, sector, and data source. Differences in N are mainly due to some items not being asked in all five data sources.

Supplement S11. Regression coefficients of policies for older workers by the proportion of older workers, separately for independent establishments and those that are part of a larger organization.

	Age distribution as categorical variables, using 50%+ as the reference category					Age distribution as continuous variables		
	0-10%	10-20%	20-30%	30-40%	40-50%	Prop 50+	Prop 50+^{2}	N
**All policies**								
Part of larger org.	-.05**	.01	.02*	.05**	.04*	.46***	-.54***	2,964
Independent est.	-.08***	-.02	-.01	.01	.01	.50***	-.57***	4,366
**Activating policies**								
Part of larger org.	.03	.05*	.04	.06*	.06	.11	-.18	2,937
Independent est.	-.00	.01	.01	.01	.01	.21**	-.32***	4,254
**Phasing out policies**								
Part of larger org.	-.08***	-.01	.02	.05**	.05*	.67***	-.67***	2,964
Independent est.	-.13***	-.04***	-.02	.01	.01	73***	-.79***	4,365

* p<.05, ** p<.01. *** p<.001

Each row represents two models, one containing the 10% groups, using 50%+ as the reference category, and one model containing proportion of older workers linearly and quadratically. All models include control variables for proportion of female workers, proportion of temporary workers, number of workers, sector, and data source.

Supplement S12. Regression coefficients for policies for older workers by the proportion of older workers, excluding establishments with fewer than 5, 10 or 15 employees, or not excluding any establishments

	Age distribution as categorical variables, using 50%+ as the reference category					Age distribution as continuous variables		
	0-10%	10-20%	20-30%	30-40%	40-50%	Prop 50+	Prop 50+^{2}	N
**All policies**								
All establishments	-.04***	.02**	.02**	.04***	.05***	.43***	-.49***	7,926
Est. with 5+ employees	-.07***	-.01	.00	.03*	.03**	.49***	-.56***	7,330
Est. with 10+ employees	-.08***	-.03**	-.01	.02*	.02*	.54***	-.58***	6,313
Est. with 15+ employees	-.08***	-.03**	-.01	.03*	.02*	.57***	-.61***	5,723
**Activating policies**								
All establishments	.02	.05***	.04**	.04**	.04**	.15**	-.24***	7,786
Est. with 5+ employees	.01	.03	.02	.03*	.03	.17**	-.27***	7,191
Est. with 10+ employees	.00	.02	.02	.03	.03	.19**	-.26**	6,246
Est. with 15+ employees	.00	.01	.02	.04*	.03	.20**	-.26**	5,656
**Phasing out policies**								
All establishments	-.08***	-.01	.02*	.05***	.05***	.64***	-.71***	7,924
Est. with 5+ employees	-.11***	-.03**	-.01*	.02*	.03	.71***	-.77***	7,329
Est. with 10+ employees	-.12***	-.05***	-.02	.02	.02	.77***	-.82***	6,312
Est. with 15+ employees	-.13***	-.05***	-.02	.02	.02	.80***	-.85***	5,722

* *p*<.05, ** *p*<.01. *** *p*<.001

Each row represents two models, one containing the 10% groups, using 50%+ as the reference category, and one model containing proportion of older workers linearly and quadratically. All models include control variables for proportion of female workers, proportion of temporary workers, number of workers, sector, and data source.

Supplement 13a. Full version of Table 4. Regression coefficients for policies for older workers by the proportion of older workers

	All policies		Activating		Phasing out		All policies		Activating		Phasing out	
	B	SE	B	SE	B	SE	B	SE	B	SE	B	SE
Proportion 50+: 50%+ (ref.)												
Proportion 50+: 0-10%	-.07***	(.01)	.01	(.02)	-.11***	(.01)						
Proportion 50+: 10-20%	-.01	(.01)	.03	(.01)	-.03**	(.01)						
Proportion 50+: 20-30%	.00	(.01)	.02	(.01)	-.01	(.01)						
Proportion 50+: 30-40%	.03**	(.01)	.03*	(.02)	.02*	(.01)						
Proportion 50+: 40-50%	.03**	(.01)	.03	(.02)	.03*	(.01)						
Proportion 50+ workers							.49***	(.04)	.17**	(.06)	.71***	(.04)
Proportion 50+ workers squared							-.56***	(.05)	-.27***	(.08)	-.77***	(.06)
Independent establishment	-.07***	(.00)	-.05***	(.01)	-.06***	(.01)	-.07***	(.00)	-.05***	(.01)	-.06***	(.01)
Number of workers	.00***	(.00)	.00***	(.00)	.00***	(.00)	.00***	(.00)	.00***	(.00)	.00***	(.00)
Female workers	-.05***	(.01)	-.00	(.02)	-.07***	(.01)	-.05***	(.01)	-.00	(.02)	-.07***	(.01)
Temporary workers	-.01	(.01)	-.00	(.02)	-.01	(.01)	-.00	(.01)	-.00	(.02)	-.01	(.01)
Sector: Services (ref.)												
Sector: Education & Science	.05***	(.01)	.08***	(.01)	.05***	(.01)	.05***	(.01)	.08***	(.01)	.05***	(.01)
Sector: Government	.15***	(.01)	.15***	(.02)	.15***	(.01)	.15***	(.01)	.15***	(.02)	.15***	(.01)
Sector: Health care	.07***	(.01)	.08***	(.02)	.08***	(.01)	.07***	(.01)	.08***	(.02)	.07***	(.01)
Sector: Manufacturing	.05***	(.01)	-.01	(.01)	.08***	(.01)	.05***	(.01)	-.01	(.01)	.08***	(.01)
Sector: Transport	-.01	(.01)	-.05**	(.02)	.01	(.01)	-.01	(.01)	-.04**	(.02)	.02	(.01)
Data source: ASPA (ref.)												
Data source: DLDP	-.06***	(.01)	.03	(.02)	-.05***	(.01)	-.06***	(.01)	.02	(.02)	-.06***	(.01)
Data source: ESWS	.05	(.03)	-.22***	(.05)	.06	(.03)	.05	(.03)	-.22***	(.05)	.05	(.03)
Data source: NES	.02**	(.01)	.29***	(.01)	-.03**	(.01)	.02*	(.01)	.29***	(.01)	-.03***	(.01)
Data source: NEWS	-.08***	(.01)	-.04***	(.01)	-.05***	(.01)	-.08***	(.01)	-.04***	(.01)	-.05***	(.01)
Constant	.36***	(.01)	.25***	(.02)	.39***	(.01)	.28***	(.01)	.26***	(.02)	.26***	(.01)
N	7,330		7,191		7,329		7,330		7,191		7,329	
R-squared	.210		.172		.171		.214		.173		.177	

* *p*<.05, ** *p*<.01. *** *p*<.001. Unstandardized regression coefficients, standard errors in parentheses.

Supplement 13b. Full version of Table 4. Regression coefficients for policies for older workers by the proportion of older workers

	Training		Flexible hours		Adaptation of tasks		Training		Flexible hours		Adaptation of tasks	
	B	SE	B	SE	B	SE	B	SE	B	SE	B	SE
Proportion 50+: 50%+ (ref.)												
Proportion 50+: 0-10%	.01	(.02)	-.01	(.04)	.02	(.02)						
Proportion 50+: 10-20%	.01	(.02)	.04	(.04)	.04	(.02)						
Proportion 50+: 20-30%	.02	(.02)	.00	(.04)	.03	(.02)						
Proportion 50+: 30-40%	.03	(.02)	.05	(.04)	.02	(.02)						
Proportion 50+: 40-50%	.03	(.02)	.03	(.04)	.01	(.03)						
Proportion 50+ workers							.16*	(.08)	.35	.19	.08	(.08)
Proportion 50+ workers squared							-.23*	(.11)	-.47	.27	-.18	(.11)
Independent establishment	-.06***	(.01)	-.07**	(.02)	-.04***	(.01)	-.06***	(.01)	-.07**	(.02)	-.05***	(.01)
Number of workers	.00***	(.00)	.00***	(.00)	.00***	(.00)	.00***	(.00)	.00***	(.00)	.00***	(.00)
Female workers	-.05*	(.02)	.06	(.05)	.03	(.02)	-.05*	(.02)	.05	(.05)	.03	(.02)
Temporary workers	-.04	(.02)	.18**	(.07)	-.00	(.02)	-.04	(.02)	.19**	(.07)	.00	(.02)
Sector: Services (ref.)												
Sector: Education & Science	.13***	(.02)	-.07*	(.03)	.08***	(.02)	.13***	(.02)	-.07*	(.03)	.08***	(.02)
Sector: Government	.10***	(.03)	.32***	(.04)	.03	(.04)	.10***	(.03)	.31***	(.04)	.03	(.03)
Sector: Health care	.13***	(.02)	.00	(.04)	.02	(.02)	.13***	(.02)	.00	(.04)	.02	(.02)
Sector: Manufacturing	-.01	(.01)	-.10***	(.03)	.03*	(.01)	-.01	(.01)	-.11***	(.03)	.03*	(.01)
Sector: Transport	-.07**	(.02)	-.03	(.06)	-.02	(.02)	-.07**	(.02)	-.03	(.06)	-.02	(.02)
Data source: ASPA (ref.)												
Data source: DLDP	.18***	(.02)					.18***	(.02)				
Data source: ESWS	-.07	(.06)					-.08	(.06)				
Data source: NES	.39***	(.02)	.19***	(.02)			.39***	(.02)	.19***	(.02)		
Data source: NEWS	.15***	(.02)			-.12***	(.02)	.15***	(.02)			-.12***	(.02)
Constant	.14***	(.03)	.41***	(.05)	.25***	(.02)	.13***	(.02)	.39***	(.04)	.27***	(.02)
N	7177		2182		4949		7177		2182		4949	
R-squared	.094		.110		.030		.094		.109		.029	

* p<.05, ** p<.01. *** p<.001. Unstandardized regression coefficients, standard errors in parentheses.

Supplement 13c. Full version of Table 4. Regression coefficients for policies for older workers by the proportion of older workers

	Part-time retirement		Reduced working hours		Early retirement		Part-time retirement		Reduced working hours		Early retirement	
	B	SE	B	SE	B	SE	B	SE	B	SE	B	SE
Proportion 50+: 50%+ (ref.)												
Proportion 50+: 0-10%	-.13***	(.02)	-.16***	(.02)	-.22***	(.03)						
Proportion 50+: 10-20%	-.06***	(.02)	-.05*	(.02)	-.11***	(.03)						
Proportion 50+: 20-30%	-.02	(.02)	-.01	(.02)	-.04	(.03)						
Proportion 50+: 30-40%	.01	(.02)	.02	(.02)	.01	(.03)						
Proportion 50+: 40-50%	.04*	(.02)	.02	(.02)	.05*	(.03)						
Proportion 50+ workers							.76***	(.07)	1.03***	(.09)	1.28***	(.13)
Proportion 50+ workers squared							-.78***	(.10)	-1.16***	(.12)	-1.36***	(.16)
Independent establishment	-.05***	(.01)	-.06***	(.01)	-.07***	(.02)	-.05***	(.01)	-.06***	(.01)	-.07***	(.02)
Number of workers	.00***	(.00)	.00***	(.00)	.00***	(.00)	.00***	(.00)	.00***	(.00)	.00***	(.00)
Female workers	-.04*	(.02)	-.06*	(.03)	-.10**	(.04)	-.04*	(.02)	-.06*	(.03)	-.10**	(.04)
Temporary workers	-.07***	(.02)	-.00	(.03)	-.06	(.04)	-.07**	(.02)	.01	(.03)	-.05	(.04)
Sector: Services (ref.)												
Sector: Education & Science	.16***	(.02)	.08***	(.02)	.12***	(.03)	.16***	(.02)	.08***	(.02)	.12***	(.03)
Sector: Government	.26***	(.03)	.18***	(.03)	.20***	(.03)	.26***	(.03)	.17***	(.03)	.20***	(.03)
Sector: Health care	.07***	(.02)	.03	(.02)	.07*	(.03)	.07***	(.02)	.03	(.02)	.08**	(.03)
Sector: Manufacturing	.10***	(.01)	.08***	(.02)	.12***	(.02)	.10***	(.01)	.08***	(.02)	.12***	(.02)
Sector: Transport	-.01	(.02)	.01	(.03)	.07*	(.04)	-.01	(.02)	.01	(.03)	.08*	(.04)
Data source: ASPA (ref.)												
Data source: DLDP	-.22***	(.02)	.06**	(.02)	-.32***	(.02)	-.23***	(.02)	.06**	(.02)	-.33***	(.02)
Data source: ESWS	.05	(.06)	.24***	(.07)	-.11	(.06)	.04	(.06)	.24***	(.07)	-.12	(.06)
Data source: NES	-.20***	(.02)	-.02	(.02)	-.26***	(.02)	-.20***	(.02)	-.02	(.02)	-.27***	(.02)
Data source: NEWS	-.21***	(.02)	.15***	(.02)			-.21***	(.02)	.15***	(.02)		
Constant	.46***	(.02)	.37***	(.03)	.59***	(.03)	.31***	(.02)	.19***	(.03)	.33***	(.03)
N	7253		7174		3361		7253		7174		3361	
R-squared	.146		.059		.186		.148		.062		.190	

* *p*<.05, ** *p*<.01. *** *p*<.001. Unstandardized regression coefficients, standard errors in parentheses.

Supplement 13d. Full version of Table 4. Regression coefficients for policies for older workers by the proportion of older workers

	Extra leave		Decrease workload		Demotion		Extra leave		Decrease workload		Demotion	
	B	SE	B	SE	B	SE	B	SE	B	SE	B	SE
Proportion 50+: 50%+ (ref.)												
Proportion 50+: 0-10%	-.18***	(.02)	-.06**	(.02)	-.00	(.01)						
Proportion 50+: 10-20%	-.04*	(.02)	.04*	(.02)	.01	(.01)						
Proportion 50+: 20-30%	-.02	(.02)	.04*	(.02)	.02	(.01)						
Proportion 50+: 30-40%	.01	(.02)	.04*	(.02)	.04**	(.01)						
Proportion 50+: 40-50%	.00	(.02)	.03	(.02)	.03*	(.01)						
Proportion 50+ workers							.97***	(.09)	.61***	(.09)	.18***	(.05)
Proportion 50+ workers squared							-.99***	(.12)	-.79***	(.11)	-.24***	(.07)
Independent establishment	-.06***	(.01)	-.04***	(.01)	-.04***	(.01)	-.06***	(.01)	-.04***	(.01)	-.04***	(.01)
Number of workers	.00***	(.00)	.00***	(.00)	.00***	(.00)	.00***	(.00)	.00***	(.00)	.00***	(.00)
Female workers	-.16***	(.02)	-.00	(.02)	.02	(.01)	-.16***	(.02)	-.00	(.02)	.02	(.01)
Temporary workers	-.09***	(.03)	.07**	(.02)	.01	(.02)	-.09***	(.03)	.07**	(.02)	.01	(.02)
Sector: Services (ref.)												
Sector: Education & Science	-.00	(.02)	.15***	(.02)	-.03**	(.01)	-.00	(.02)	.14***	(.02)	-.03**	(.01)
Sector: Government	.15***	(.03)	.05	(.03)	.04*	(.02)	.14***	(.03)	.04	(.03)	.04*	(.02)
Sector: Health care	.10***	(.02)	.06**	(.02)	.00	(.01)	.10***	(.02)	.06**	(.02)	.00	(.01)
Sector: Manufacturing	.17***	(.02)	.12***	(.01)	-.02*	(.01)	.17***	(.02)	.12***	(.01)	-.02	(.01)
Sector: Transport	.00	(.02)	.09***	(.02)	-.03*	(.01)	.00	(.02)	.09***	(.02)	-.03*	(.01)
Data source: ASPA (ref.)												
Data source: DLDP	.17***	(.02)	.00	(.02)	.04**	(.01)	.16***	(.02)	-.00	(.02)	.04**	(.01)
Data source: ESWS	.24***	(.07)	-.01	(.07)	.24***	(.04)	.24***	(.07)	-.02	(.07)	.24***	(.04)
Data source: NES	.05*	(.02)	-.10***	(.02)	.05***	(.01)	.04	(.02)	-.11***	(.02)	.05***	(.01)
Data source: NEWS	.05**	(.02)	-.04*	(.02)	.02	(.01)	.05**	(.02)	-.04*	(.02)	.02	(.01)
Constant	.57***	(.03)	.28***	(.03)	.06***	(.02)	.38***	(.03)	.22***	(.02)	.05***	(.02)
N	7184		7178		7173		7184		7178		7173	
R-squared	.097		.035		.051		.099		.035		.050	

* *p*<.05, ** *p*<.01. *** *p*<.001. Unstandardized regression coefficients, standard errors in parentheses.

Supplement 13e. Full version of Table 4. Regression coefficients for policies for older workers by the proportion of older workers

	Ergonomic measures		Irregular hours		Ergonomic measures		Irregular hours	
	B	SE	B	SE	B	SE	B	SE
Proportion 50+: 50%+ (ref.)								
Proportion 50+: 0-10%	-.04	(.02)	-.10***	(.02)				
Proportion 50+: 10-20%	-.01	(.02)	-.03	(.02)				
Proportion 50+: 20-30%	-.02	(.02)	-.01	(.02)				
Proportion 50+: 30-40%	.02	(.02)	.04	(.02)				
Proportion 50+: 40-50%	.02	(.03)	.03	(.02)				
Proportion 50+ workers					.33***	(.09)	.71***	(.08)
Proportion 50+ workers squared					-.39**	(.12)	-.78***	(.10)
Independent establishment	-.08***	(.01)	-.08***	(.01)	-.08***	(.01)	-.08***	(.01)
Number of workers	.00***	(.00)	.00***	(.00)	.00***	(.00)	.00***	(.00)
Female workers	-.06*	(.03)	-.13***	(.02)	-.06*	(.03)	-.13***	(.02)
Temporary workers	-.07**	(.03)	.05*	(.02)	-.07**	(.03)	.06*	(.02)
Sector: Services (ref.)								
Sector: Education & Science	.02	(.02)	-.07***	(.02)	.01	(.02)	-.07***	(.02)
Sector: Government	.21***	(.03)	.17***	(.03)	.21***	(.03)	.16***	(.03)
Sector: Health care	.08***	(.02)	.23***	(.02)	.07**	(.02)	.22***	(.02)
Sector: Manufacturing	.04*	(.02)	.08***	(.01)	.04*	(.02)	.08***	(.01)
Sector: Transport	-.04	(.03)	.07***	(.02)	-.04	(.03)	.07***	(.02)
Data source: ASPA (ref.)								
Data source: DLDP	-.33***	(.07)			-.32***	(.07)		
Data source: ESWS	.20***	(.02)	.04	(.02)	.20***	(.02)	.03	(.02)
Data source: NES	-.14***	(.02)	.00	(.02)	-.14***	(.02)	.00	(.02)
Data source: NEWS			.01	(.02)			.01	(.02)
Constant	.46***	(.03)	.27***	(.02)	.41***	(.02)	.15***	(.02)
N	6111		7099		6111		7099	
R-squared	.143		.100		.144		.102	

* $p<.05$, ** $p<.01$. *** $p<.001$. Unstandardized regression coefficients, standard errors in parentheses.
